# Evaluation of Steels Susceptibility to Hydrogen Embrittlement: A Thermal Desorption Spectroscopy-Based Approach Coupled with Artificial Neural Network

**DOI:** 10.3390/ma13235500

**Published:** 2020-12-02

**Authors:** Evgenii Malitckii, Eric Fangnon, Pedro Vilaça

**Affiliations:** Department of Mechanical Engineering, School of Engineering, Aalto University, 02150 Espoo, Finland; eric.a.fangnon@aalto.fi (E.F.); pedro.vilaca@aalto.fi (P.V.)

**Keywords:** thermal desorption spectroscopy, hydrogen embrittlement, hydrogen sensitivity, steels, artificial neural network

## Abstract

A novel approach has been developed for quantitative evaluation of the susceptibility of steels and alloys to hydrogen embrittlement. The approach uses a combination of hydrogen thermal desorption spectroscopy (TDS) analysis with recent advances in machine learning technology to develop a regression artificial neural network (ANN) model predicting hydrogen-induced degradation of mechanical properties of steels. We describe the thermal desorption data processing, artificial neural network architecture development, and the learning process beneficial for the accuracy of the developed artificial neural network model. A data augmentation procedure was proposed to increase the diversity of the input data and improve the generalization of the model. The study of the relationship between thermal desorption spectroscopy data and the mechanical properties of steel evidences a strong correlation of their corresponding parameters. A prototype software application based on the developed model is introduced and is openly available. The developed prototype based on TDS analysis coupled with ANN is shown to be a valuable engineering tool for steel characterization and quantitative prediction of the degradation of steel properties caused by hydrogen.

## 1. Introduction

Hydrogen embrittlement (HE) is often designated as the reason for the unexpected failure of engineering components exposed to hydrogen during manufacturing or in service. Failure of a bolted joint led to an offshore spill of approximately 400 barrels of drilling fluids, in late 2012, that was determined to be induced by environmental HE with the contribution of inappropriate use of zinc plating and improper cathodic protection [1]. Corrosion-induced HE in boiler tubes and water wall tubes was found to be a significant problem in the steam-power cogeneration plants initiated by under deposit corrosion attack [2,3]. High-temperature hydrogen attack (HTHA) is an issue causing heat exchanger failures in the gas and oil industry [4,5] by hydrogen dissociating and dissolving in steels reacting with carbon and forming methane. The HTHA mechanism is different from the one driving the low-temperature hydrogen-induced damage, but it is still controlled by hydrogen diffusion. Control of the degradation of steel properties caused by hydrogen is a complex task, since the hydrogen interaction with steels depends on microstructural properties of the materials, stress state, and environmental conditions controlling the hydrogen uptake [6,7]. Studies of HE in carbon steels have often shown the presence of a certain threshold of hydrogen concentration affecting the steel fracture mode [3,8,9]. This effect, however, depends strongly on the microstructure. For example, it does not show the same influence in duplex stainless steels where the rate of HE increases smoothly with the increase of the hydrogen concentration [10]. Djukic et al. proposed a unified model to assess HE by combining phenomenological research of hydrogen–metal interactions with a predictive model based on in-situ hydrogen concentration measurement [3]. Another way to evaluate the macroscopic mechanical response (namely, ultimate tensile strength, yield stress, and elongation to fracture) was proposed by Thankachan et al. for aluminum alloys using artificial neural network (ANN), creating a strong relationship between the input data (chemical composition, strain rate, and hydrogen charging conditions) and the targeted performance parameters of mechanical response. In addition, a strong correlation between steel hardness/strength and HE susceptibility was defined for individual steels after different heat treatment procedures [11,12,13]. The second-order effect on HE susceptibility was attributed to several factors, including chemical composition and heat treatment, that control the microstructure of the materials. These factors ultimately affect hydrogen diffusion and trapping [11]. Nevertheless, systematic research of hydrogen–metal interactions in different alloys is important for the development of a robust modeling approach for in-situ assessment and prediction of HE in actual engineering applications [3,13,14].

**Figure 1:**
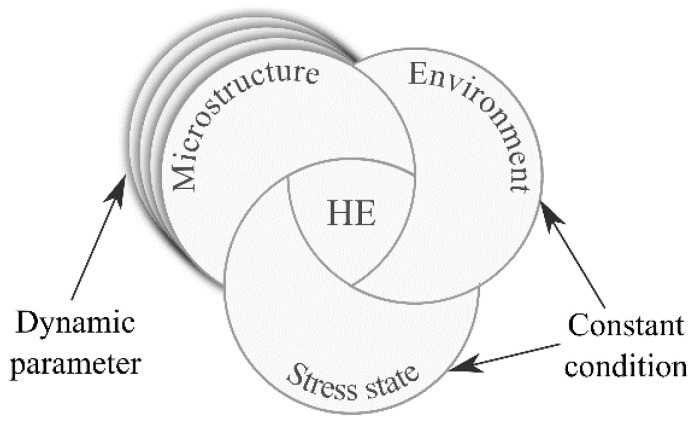
The overview of the conditions considered as the parameters controlling the hydrogen embrittlement in steels in the developed approach.

With the use of recent advances in machine learning technologies, we have developed and published a first conceptual approach in the prediction of HE susceptibility of steels correlating the macroscopic mechanical property changes, caused by hydrogen, with hydrogen thermal desorption spectroscopy (TDS) of steels [15]. TDS spectroscopy provides indirect assessment of the material microstructural features controlling the hydrogen diffusivity, solubility, and trapping of hydrogen in the studied steel. The correlation between hydrogen thermal desorption characteristics and material microstructural features has been under the active study in recent years [16,17,18,19]. The assessment of creep damage in heat-resistant ferritic stainless steel using thermal desorption analysis was proposed by Yamashita et al., evidencing a clear overall TDS shape variation with creep, depending on the test conditions [20]. The microstructural change associated with formation of non-metallic inclusion caused by the heat treatment procedure was studied in low-carbon ferritic steel showing the effect of vanadium carbide effective surface area on hydrogen trapping [21]. A model of TDA was proposed based on a correlation between the trap site density as a function of the effective surface area of the vanadium carbides [21]. Investigation of hydrogen trapping in lab cast Fe–C–W alloys using TDS evidenced the presence of the peaks correlated to W carbides of different sizes and compositions [22]. Worth noting is that the impact of dislocation density on hydrogen trapping capacity is a very common observation [18,19,20,21]. The parameters of stress state and environmental conditions controlling the hydrogen uptake were defined as constants at this particular stage of the research (see Figure 1). The approach was tested with different ANN model architectures evidencing a strong correlation between TDS of hydrogen and the target value of the hydrogen sensitivity parameter (HSP) standing as a measure of HE susceptibility of steels [15]. The objective of the present paper is to discuss the benefits of the approach and introduce a novel prototype software application for prediction of HSP of steels based on the recently developed ANN model. The results are evaluated considering the TDS data processing, ANN architecture development, and machine learning process towards quality prediction. The approach will promote a safer structural performance, contributing to a more sustainable operation of key engineering systems with high environmental, societal and economic impact, such as power plants.

## 2. Materials and Methods

### 2.1. Sample Preparation and Mechanical Testing

Steels with different microstructures were chosen for this study to ensure variation in hydrogen susceptibility. The chemical compositions of the studied steels are shown in Table 1. The chemical compositions of the studied high-strength steels are the same, while the microstructures and mechanical properties are different due to different heat treatment procedures applied during the manufacturing process. Microstructures of the studied ferritic–martensitic high-strength steel (FMHSS) grades and corresponding heat treatment procedures were described in detail by Hickel et al. [13]. Tensile specimens with a gauge length of 32 mm and a width of 5 mm were cut by electrical discharge machining from the 1 mm thickness steel sheets parallel to its rolling direction. The gauge length part of the specimens was mechanically polished with No.1200 emery paper.

Constant extension rate tensile (CERT) tests were performed with a 35 kN MTS desktop machine (MTS Systems Corporation, Eden Prairie, MN, USA) at a strain rate of 10^−4^ s^−1^. Tensile specimens were tested in as-supplied condition and during continuous hydrogen charging. The hydrogen charging was performed electrochemically using the 1N H_2_SO_4_ solution with 20 mg∙L^−1^ of CH_4_N_2_S at the potential of −1.08 V, −1.2 V, and −1.225 V for austenitic stainless steel (ASS), ferritic stainless steel (FSS), and FMHSS grades, respectively. Specimens tested during continuous hydrogen charging were pre-charged to approach the homogeneous hydrogen distribution through the specimen thickness for 72 h, 2 h, and 1 h for ASS, FSS, and FMHSS grades, respectively.

The hydrogen sensitivity parameter (HSP) was calculated from the reduction of elongation to fracture as follows:

(1) $$\mathrm{HSP}=\frac{\left(\varepsilon -\varepsilon_{H}\right)}{\varepsilon }\times 100\%$$

where ε is the elongation to fracture of the specimens tested in the as-supplied condition, and $\varepsilon_{H}$ is the elongation to fracture of the H-charged specimen.

### 2.2. Hydrogen Thermal Desorption Spectroscopy

Hydrogen was measured using a thermal desorption spectroscopy (TDS) (Aalto University, Espoo, Finland) apparatus developed at Aalto University Department of Mechanical Engineering, Finland. The TDS apparatus consists of an ultra-high vacuum (UHV) measurement chamber equipped with a vacuum furnace and mass spectrometer. The UHV chamber was coupled with the air-lock chamber, where the specimen was introduced first, and pre-pumped to an intermediate pressure of 2 × 10^−5^ mbar. After the intermediate pressure was achieved, the specimen was automatically transported to the UHV chamber using the specimen transport system. Measurements were performed under ultra-high vacuum conditions starting from 8 × 10^−8^ mbar. The hydrogen desorption rate was measured in the temperature range from room temperature (RT) to 1070 K with a linear heating rate of 10 × K∙min^−1^. Before measurement, the specimens were cleaned by acetone in an ultrasonic bath for 1 min and dried under helium gas flow to remove the water residuals from the specimen surface. The characteristic size of a TDS specimen is 1 × 5 × 15 mm^3^. Steel samples were measured in the as-supplied condition. Thus, only the metallurgical hydrogen accumulated into the steels during the manufacturing and storage was considered to address the steel microstructural response using hydrogen as a probe.

### 2.3. Measurement Data Processing

**Figure 2:**
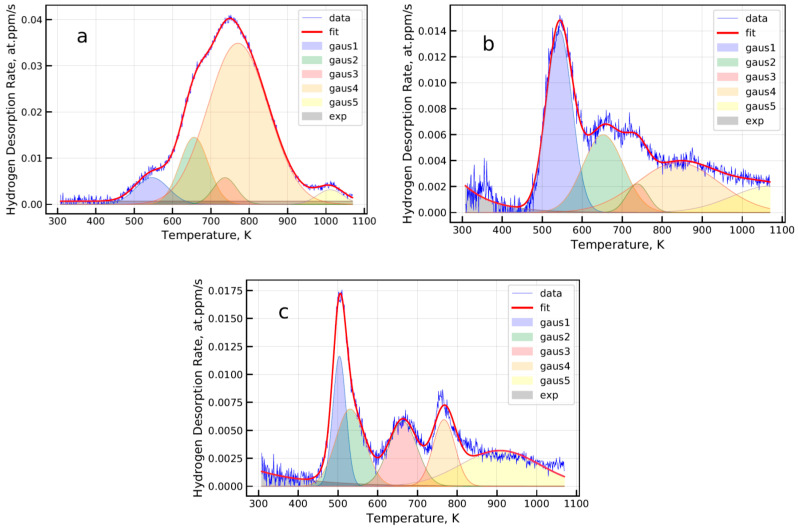
Examples of the thermal desorption spectroscopy of hydrogen from the as-supplied (**a**) austenitic stainless steel, (**b**) ferritic stainless steel, and (**c**) ferritic–martensitic high-strength steel. Spectroscopy curves are fitted with the sum of five Gaussian peaks and exponential background.

Fitting of the spectroscopy data was performed using five Gaussian peaks and exponential functions (see Figure 2). The equation of the Gaussian peak was introduced to the fitting algorithm as follows:

(2) $$f\left(x\right)=a\times \exp^{-\frac{1}{2}\times \frac{{\left(x-b\right)}^{2}}{c^{2}}}$$

where a, b, and c are fitting parameters defining the peak amplitude, peak temperature position, and width, respectively.

Fitting parameters of the Gaussian peaks were collected, successively forming the TDS spectra descriptor. The experimental dataset of the TDS spectra descriptors can be generalized as follows:

(3) $$\begin{array}{ccccc} x_{11} & x_{21} & x_{31} & \cdots & x_{i1}\\ x_{12} & x_{22} & x_{32} & \cdots & x_{i2}\\ x_{13} & x_{23} & x_{33} & \cdots & x_{i3}\\ \vdots & \vdots & \vdots & \ddots & \vdots \\ x_{1j} & x_{2j} & x_{3j} & \cdots & x_{\mathrm{ij}} \end{array}$$

where $x_{\mathrm{ij}}$ is the descriptor parameter, i is the descriptor number from 1 to 15, and j defines the number of spectra. Descriptor numbers from 1 to 3 corresponded to the first Gaussian peak parameters defining the peak amplitude, peak temperature position, and width, respectively. The next group of three parameters corresponded to the next Gaussian peak and so on. The experimental dataset comprised 50 descriptor lines (j = [1, 50]) of 15 parameters each. The diversity of data was increased by the generation of an artificial dataset complementing the experimental dataset. The parameters of the first Gaussian peak were chosen randomly from the uniform distribution between the minimum and maximum parameter values of the experimental dataset. The parameters of the other four Gaussian peaks were generated according to their correlation with the parameters of the first Gaussian peak for each steel grade separately. The algorithm of artificial data generation can be summarized as follows:

(4) $$\begin{array}{c} i=\left[1,\;3\right]\to {\bar{x}}_{i}\;\sim \cup^{\;}\left(\min\left(x_{i}\right),\max\left(x_{i}\right)\right)\\ i=\left[4,\;15\right]\to \;\{\begin{array}{cc} {\bar{x}}_{i}\;\sim \cup^{\;}\left(\min\left(x_{i}\right),\max\left(x_{i}\right)\right) & \mathrm{if},\;\mathrm{Corr}\left(x_{i\;=\;\left[1,3\right]},x_{i\;=\;\left[4,15\right]}\right)\leq 0.6\\ {\bar{x}}_{i}=F\left(x_{i\;=\;\left[1,3\right]}\right) & \mathrm{if},\;\mathrm{Corr}\left(x_{i\;=\;\left[1,3\right]},x_{i\;=\;\left[4,15\right]}\right)>0.6 \end{array} \end{array}$$

where ${\bar{x}}_{i}$ is the descriptor parameter of the artificial spectroscopy data. $F\left(x_{i}\right)$ is a linear function defined by the fitting of the relationship between $x_{i\;=\;\left[1,3\right]}$ and $x_{i\;=\;\left[4,15\right]}$. It is worth noting that the Pearson correlation coefficients between only the same type of descriptor parameters defining the peak height, temperature position, and width were considered.

The described data augmentation procedure allowed the experimental dataset to be complemented with artificial TDS fitting parameters. A dataset with 20,000 TDS samples was generated comprising 2000 TDS samples for each steel. A normalization procedure is mandatory for the input data of the artificial neural network model [14,23]. A normalization procedure was applied individually for each fitting parameter, dividing each element in the column of the dataset matrix (see Equation (3)) by the maximum value from the column. Then, the full dataset was split into training, validation, and test datasets. The training dataset comprised 6000 TDS samples of six steels (AISI 304, AISI 441, VA1000_TM05, VA1400_TM, and two batches of VA1400_MTM steel) from the list of the studied materials with the corresponding targets of the HSP-value measured experimentally. The validation dataset comprised 4000 data pairs of the remaining four steels (AISI 409, VA1000_M05, VA1200_MTM, and one batch of VA1400_MTM steel). The test dataset comprised 10,000 data pairs of all the studied steels, where each studied steel contained 1000 data pairs.

### 2.4. Regression Artificial Neural Network Modeling

**Figure 3:**
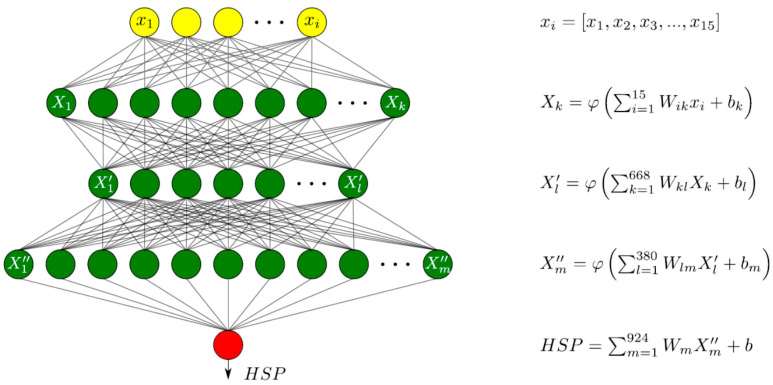
Deep feedforward artificial neural network architecture. Input layer, output layer, and hidden layer nodes are marked in yellow, red, and green colors, respectively. The corresponding outputs of the layer nodes are shown at the right side of the image. The input data of the nodes was multiplied with the weights (W), and their summation obtained an addition of bias (b ). The weights and bias transformed the input data linearly. The non-linear transformation was performed by the rectified linear unit (ReLU) activation function (φ ) defined as f(x)=max(0,x).

Deep feedforward architecture was considered for the development of the regression ANN model [15]. The architecture of the developed model is schematically shown in Figure 3. The input of the ANN model was the row vector of the normalized TDS fitting parameters, as described in the “measurement data processing” section. The size of the input vector was 15, which corresponded to the number of fitting parameters. The hidden layer comprised three densely connected layers of 668, 380, and 924 neurons (see Figure 3). A single neuron is defined by the input data multiplied by the weights (W), and their summation obtains an addition of bias (b). The weights and bias transform the input data linearly. The non-linear transformation is performed by the rectified linear unit (ReLU) activation function (φ), defined as f(x)=max(0,x) [24]. The ANN ends with the output layer comprising a single neuron without an activation function [23].

The ANN learning process was performed by the RMSprop adaptive learning rate method, a form of stochastic gradient descent proposed by Geoff Hinton [25]. The mean squared error (MSE) between the experimental and predicted HSP-values was considered as a loss score for application in the learning process of the developed ANN model (see Equation (5)), where n is a number of predictions, $Y_{i}$ is the experimental target data, and ${Y^{\prime }}_{i}$ is the predicted output by the ANN model.

Mean absolute error (MAE) is a common regression metric that allows the performance of the regression ANN model to be evaluated (see Equation (6)) [14,23]. Hyper-parameters of the ANN model such as the number of neurons in the hidden layer and number of layers were optimized first using MAE-based validation by the trial-and-error method, since the general-purpose method for definition of ANN topology did still not exist. Final tuning of the hyper-parameters was performed using the random search algorithm [26]. The minimum MAE was obtained by training the ANN model with about 150 training iterations (epochs). Best model parameters (weights and bias values) corresponding to the minimum of MAE at the validation dataset were carried out using the early stopping of the learning process.

(5) $$\mathrm{MSE}=\frac{1}{n}\sum_{i=1}^{n}{\left(Y_{i}-{Y^{\prime }}_{i}\right)}^{2}$$

(6) $$\mathrm{MAE}=\frac{1}{n}\sum_{i=1}^{n}\left|{Y^{\prime }}_{i}-Y_{i}\right|$$

Development, training, validation, and testing of the ANN model were performed in Python programming language using Keras open-source neural network library running on top of TensorFlow software for machine learning applications.

## 3. Results

**Figure 4:**
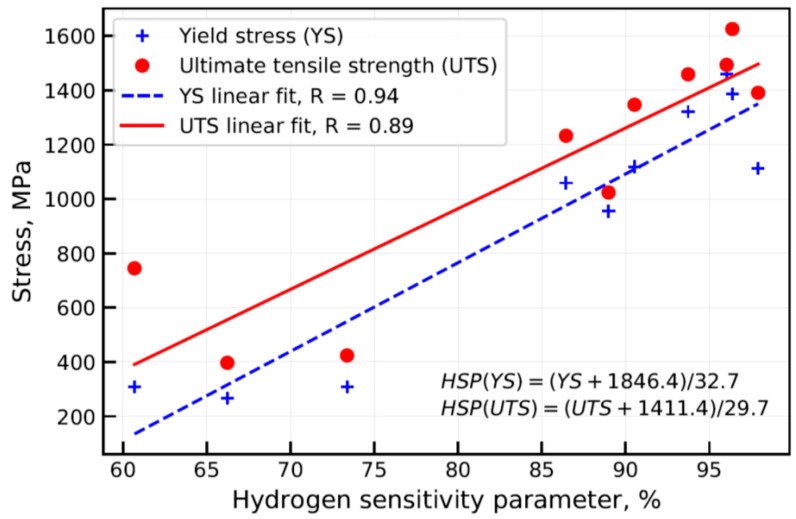
Relationship between yield stress (YS) and ultimate tensile strength (UTS) vs. hydrogen sensitivity parameter (HSP) measured experimentally.

The effect of electrochemical hydrogen charging on the elongation to fracture of the studied steels and value of the hydrogen sensitivity parameter (HSP) are summarized in Table 2. The relationship between yield stress (YS), ultimate tensile strength (UTS), and HSP were studied, evidencing an increase of the HSP with increases of the YS and UTS of the studied steels (see Figure 4). Linear regression (LR) was applied to model the relationship between the YS, UTS, and HSP. Pearson coefficient of correlation (R) was calculated between the HSP measured experimentally and those calculated using the linear regression model for YS and UTS data to be about 0.94 and 0.89, respectively. The MAE of the linear regression model was calculated for the YS and UTS data to be about 3.9% and 5.5%, respectively. Standard deviation (STD) was found to be about 2.2% and 3.5% for YS and UTS data, respectively. 

Tensile toughness was calculated for the specimens tested in as-supplied ($K_{\mathrm{Ic}}$) and H-charged ($K_{\mathrm{IH}}$) conditions. The results are summarized in Table 2. In the presence of hydrogen charging, hydrogen-induced cracks developed and propagated well below the $K_{\mathrm{Ic}}$. The subcritical threshold value of tensile toughness in the presence of hydrogen could be calculated from about 5% to 38% of $K_{\mathrm{Ic}}$.

**Figure 5:**
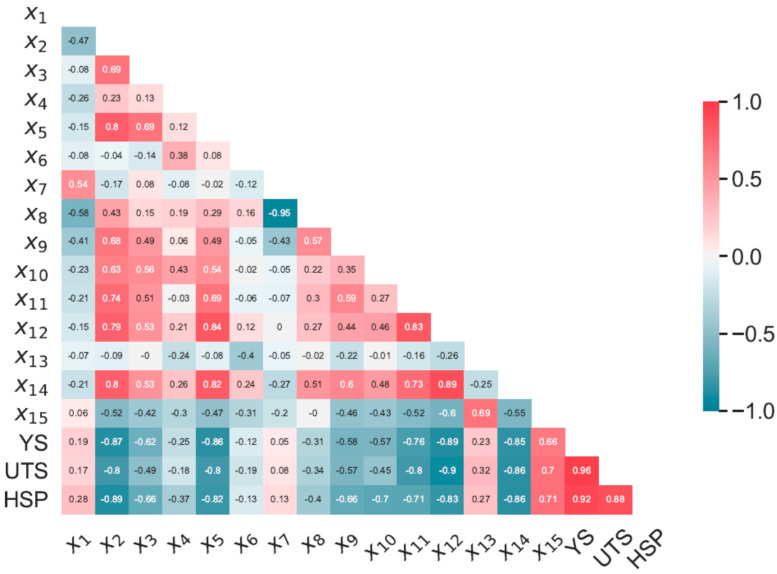
R-value calculated between the thermal desorption spectroscopy (TDS) curve-fitting parameters of the studied steels measured experimentally including yield stress (YS), ultimate tensile strength (UTS), and hydrogen sensitivity parameter (HSP) data.

Thermal desorption spectroscopy (TDS) curve-fitting parameters were defined according to the measurement data processing approach, as described in the Methods section. R-value was calculated between the TDS curve-fitting parameters of the studied steels measured experimentally and summarized within the matrix plot, as shown in Figure 5. In addition, the matrix plot was complemented with R-values calculated between YS, UTS, and TDS curve-fitting parameters. The fitting parameters ($x_{1},{\;x}_{2},{\;x}_{3},\;\cdots ,{\;x}_{15}$) corresponded to the amplitude, peak temperature position, and width of the fitted Gaussian curves, successively, as described in the Methods section. According to the rule of thumb, the relationship exists if $\left|R\right|\geq 2/\sqrt{n}$, where n is the number of samples [27]. This threshold value was calculated to be about |R|= 0.63. Figure 5 depicts a good correlation between YS, UTS and $x_{2}$, $x_{5}$, $x_{14}$ parameters corresponding to the temperature position of the first, second, and fifth Gaussian peak calculated to be |R|> 0.8. A similar rate of correlation could be observed between some of the fitting parameters such as $x_{2}$ and $x_{5}$, $x_{2}$ and $x_{14}$, $x_{5}$ and $x_{12}$, etc. There were, however, parameters with relatively low strength of the cross-correlation such as $x_{1}$, $x_{4}$, and $x_{6}$. It is worth mentioning that the YS and UTS had the best correlation with parameter $x_{12}$, corresponding to the width of the fourth Gaussian peak. Correlation of the YS and UTS with Gaussian peak width was not as systematic as that observed for temperature peak positions. The correlation analysis of the fitting parameters with the HSP showed similar results, evidencing the best rate of correlation between HSP and $x_{2}$, $x_{5}$, $x_{14}$, and $x_{12}$.

**Figure 6:**
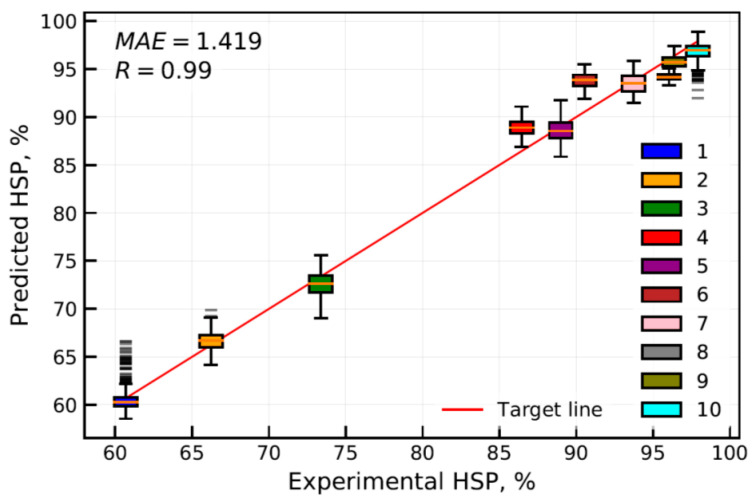
Correlation between the experimental and predicted HSP at test dataset. Box plot numbers are associated with the studied steel grades according to Table 1.

**Figure 7:**
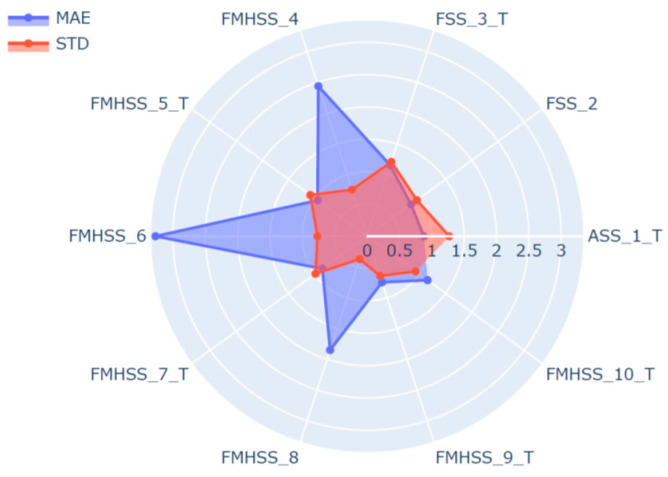
MAE and STD of predicted HSP calculated for individual steel. Labels of the plot denote steel type and ordinal number of the studied steels according to the Table 1. Steel grades presented in the training dataset are marked by the letter T. The dimension is in %.

The regression ANN model (depicted in Figure 3) was developed and trained according to the methodology described in the Methods section. The ANN model was trained at the training dataset comprising six steel grades and 6000 data pairs. This provided training data covering a large domain of the targeted structural steels. During training, the developed model was validated at the validation dataset comprising four additional steel grades and 4000 data pairs to find the optimal ANN model parameters corresponding to the minimum of MAE calculated at the validation dataset. After the training was complete, the HSP targets were predicted at the test dataset comprising 1000 data pairs for each studied steel to evaluate the accuracy of the developed ANN model. The test dataset included TDS fitting parameters that were not exposed during ANN model training and validation, that is essential for ANN model accuracy assessment. Worthy of note is that the test dataset contained the artificial TDS fitting parameters produced by the data augmentation procedure using the same algorithm as for training and validation datasets, which may have resulted in some data leakage affecting the accuracy assessment. R-value calculated between the experimental and predicted HSP-values showed a linear correlation of 0.99 at the test dataset (see Figure 6). MAE of the HSP prediction using the developed ANN model at the test dataset was calculated to be about 1.4%. The advantage of the proposed measurement data processing is obvious considering our previous study, where MAE was calculated to be about 2.8% and 4.5% for feed-forward and convolutional neural network architectures, respectively [15]. The detailed study of the prediction error calculated at the test dataset for individual steels showed some increase of MAE calculated for steels included in the validation compared to those used in the model learning process (see Figure 7). The standard deviation of the predicted HSP did not vary significantly between the different steels, evidencing, however, some reduction in deviation of the HSP prediction at steel grades included in the validation dataset.

## 4. Discussion

**Figure 8:**
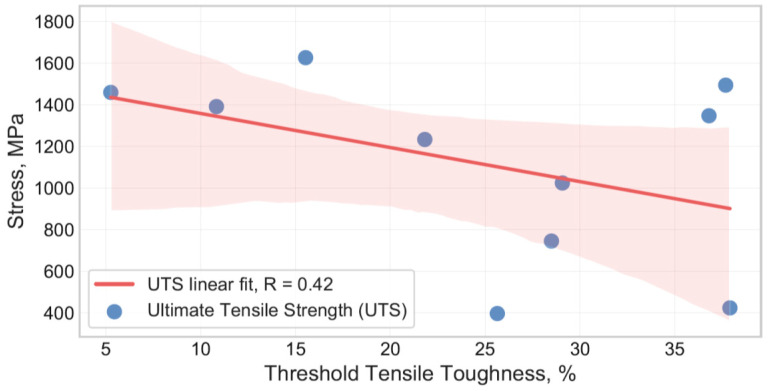
Threshold tensile toughness ($(K_{\mathrm{IH}}\cdot 100)/K_{\mathrm{Ic}}$) as a function of the ultimate tensile strength (UTS) calculated for the studied steels. Translucent bands around the regression line define the confidence interval of the regression.

Susceptibility of steels to HE increases with an increase in material strength. This relationship was extensively studied evidencing that material conditions such as strength and/or hardness are primary conditions defining the susceptibility of steels to HE [11,12,13]. The results, as presented in Figure 4, confirm the statement showing a certain tendency between UTS, YS, and HSP of the studied steel grades. The linear regression modeling reveals a comparable HSP prediction quality at YS and UTS data for the studied steels, caused apparently by its high cross correlation rate, as shown in Figure 5. Brahimi et al. [11] showed the embrittlement ratio decreasing smoothly with an increase in the hardness of individual high-strength steel grades. However, different alloys can have different hydrogen sensitivities despite the similar properties of hardness/strength that complicate the accurate prediction of changes in the steel properties in the presence of hydrogen [11,13]. Such a phenomenon was attributed to a combination of factors such as chemical composition, heat treatment, aging conditions affecting the microstructure of steels, and ultimately, hydrogen transport and trapping. ANN model input data comprising chemical composition together with hydrogen charging parameters were found to be sufficient for effective prediction of YS, UTS, and ε degradation of aluminum alloys, as shown by Thankachan et al. [14]. In order to evaluate the cumulative effect of hydrogen on both stress and strain of the studied steels, the tensile toughness was calculated for steels tested in as-supplied and hydrogen charged conditions (see Table 2). Threshold tensile toughness was designated through $K_{\mathrm{IH}}$ as a percentage of $K_{\mathrm{Ic}}$. Figure 8 evidences the threshold tensile toughness decreasing with the increase of the UTS of the steels. However, the deviation of the results was found to be significant for the studied batch of the specimens. One can conclude that considering the cumulative effect of hydrogen on both plasticity and strength of the studied steels the UTS is insufficient for evaluation of susceptibility of steels to hydrogen embrittlement. Quantitative assessment of the effect of hydrogen on steel toughness is of the utmost importance for the characterization of the material’s resistance to fracture and effective modeling of crack propagation in the presence of hydrogen. The characterization and modeling of a joint effect of microstructure and hydrogen on change of the steel toughness using machine learning technology is the objective of future research.

Ebihara et al. proposed a fundamental model for the reproduction of the total thermal desorption spectra of hydrogen based on the mass conservation of hydrogen in the specimen with the activation energy law for the diffusion, trapping, and detrapping processes [27,28,29,30,31]. This model is applied to simulate complex thermal desorption spectra of hydrogen with more than one peak involved [27,29]. The experimental study of correlation between the steels’ microstructure and thermal desorption spectroscopy of hydrogen reveals a certain hydrogen trapping change as a function of microstructural defects and its density [16,17,18,19,20,21]. From above, one can assume that the hydrogen thermal desorption spectra from steels and alloys incorporate the information on hydrogen diffusion and trapping controlled by the material microstructure. Figure 5 shows a strict correlation between the first, second, and fifth peak temperature positions ($x_{2}$, $x_{5}$, $x_{14}$) and fourth peak width ($x_{12}$) with YS and UTS properties of the studied steels. From above, one can assume the TDS of hydrogen incorporates the primary parameters defining the HE susceptibility of the studied steels. These fitting parameters shows also a high rate of correlation with HSP and play apparently the most important role in ANN model training. At the same time, some TDS curve-fitting parameters, such as $x_{1}$, $x_{4}$, $x_{6}$, $x_{7}$, $x_{8}$, and $x_{13}$, have a weak relationship with the YS, UTS, HSP, and $x_{2}$, $x_{5}$ accordingly. However, these parameters contribute apparently to the HSP prediction quality of the studied steels. It is worth noting that the studied linear correlation between the TDS curve-fitting parameters does not describe the causation completely, and the unrelated parameters may have a complex mathematical relationship.

The regression feedforward ANN model was designed to take a complete dataset of the TDS curve fitting parameters as the input data to predict the value of HSP of the studied steels. The average MAE of the ANN model surpassed markedly that which was calculated for the LR model, despite the average MAE of LR model showing a relatively small error at the experimental dataset. However, the prediction quality of the ANN and LR models can change significantly with an increase of diversity of the experimental data. The detailed analysis of MAE calculated for individual steels (see Figure 7) from the test dataset reveals a perceptible increase of MAE at steel grades chosen for validation of the ANN model during the learning process. The highest absolute error of HSP prediction was calculated to be about 5.9% for AISI 304 and VA1400MTM steel grades representing the steel grades with the lower and higher hydrogen sensitivity parameters, respectively. The observed variation can be decreased apparently by the increase of diversity of steels chosen for the ANN model learning process. In addition, one can observe that the standard deviation increased as the HSP prediction came closer to its target value (see Figure 7). The observed effect relates apparently to the bias-variance problem that describes the challenge to minimize simultaneously both sources of error in a supervised learning algorithm. Thus, minimizing bias is accompanied usually with an increase of variance and vice versa. Proper regularization techniques may considerably improve the machine learning regression algorithm performance. For an ANN, the increase of the hidden layer width may reduce the total error caused by bias and variance [32].

**Figure 9:**
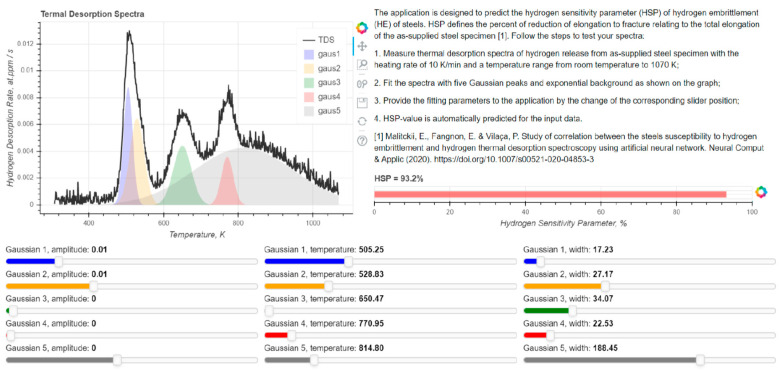
Prototype interactive application predicting the HSP using the developed ANN model. Parameters of the spectra can be adjusted using sliders. The value of the HSP changes on-line with the change of the input parameters of the TDS spectra. The developed model was deployed and it is openly available online [33].

The developed ANN model input data includes all the TDS curve-fitting parameters that are probably excessive due to the relatively high cross correlation of some of them (see Figure 5). Feature selection and optimization of the input data is the aim of future research. Nevertheless, the developed ANN model provides the proper prediction of the HSP of steels of different microstructures, the TDS data of which lies under the probabilistic distribution of the training data. TDS of hydrogen incorporates the information on microstructure, hydrogen transport, and trapping of the studied steels that contribute markedly to the ability of the ANN model to predict the degradation of the mechanical properties caused by hydrogen. Presently human supervision of the prediction is needed to prevent the misuse of the developed approach caused by steel conditions, hydrogen distribution, post-processing of the TDS curves, etc. We believe that with further development of the experimental database and improvement of the prediction quality, the approach will contribute to the fracture mechanics analysis of steel engineering components in the presence of sources of hydrogen. Such an engineering tool can markedly improve safety and reduce the time and labor cost of the steel examination. The developed model was deployed, and it is openly available for testing as a prototype web application with interactive control of the input hydrogen TDS fitting parameters and automatic calculation of the HSP (see Figure 9) [33]. New releases of the web application and ANN model are coming in the future with the development of the research.

## 5. Conclusions

The artificial neural network model predicting the susceptibility to hydrogen parameter was developed. The model was trained using a dataset of TDS curve fitting parameters measured from different steel grades. TDS was treated as a parameter of the microstructure of the studied steel, while the stress state and environmental conditions were assumed to be constant due to the same approach in tensile testing and hydrogen charging procedures applied for austenitic, ferritic, and ferritic–martensitic steel grades, respectively. The HSP was calculated from the reduction of the plasticity of the studied steels caused by hydrogen charging. The ANN model provides a reliable prediction of the HSP calculated from the reduction of elongation to fracture for steels of different microstructures, the TDS data of which lies under the probabilistic distribution of the trained data. MAE of the HSP prediction using the developed ANN model at the test dataset was calculated to be about 1.4%. Worth noting is that the measurement of plasticity change caused by hydrogen is a widely used approach for HSP calculation; however, considering the effect of hydrogen on both strength and plasticity by calculation of the threshold toughness, a cumulative analysis of the hydrogen embrittlement effect is required for reliable fracture mechanics computational modeling. The approach shows promising results in the prediction of the hydrogen embrittlement effect on elongation at fracture, promoting safer structural performance and contributing to a more sustainable operation of engineering systems.

## 6. Patents

A patent application for the described approach was filed to the Finnish Patent and Registration Office (FI 20197123). 

## Figures and Tables

**Table 1:** Chemical composition of the steels selected for training and validation of the artificial neural network (ANN) models (wt.%).

Steel\Type\Content		C	Si	Mn	Ti	Mo	Cu	Cr	Ni	Nb	N	P	S
AISI 304	ASS	0.049	0.43	1.49	-	-	0.43	18.2	8.2	-	0.047	-	-
AISI 409	FSS	<0.03	<1	<1	0.26	-	-	11.75	0.5	-	-	<0.045	0.025
AISI 441	FSS	0.014	0.61	0.42	0.138	0.024	0.12	17.7	0.2	0.393	-	0.03	<0.015
VA1000_M05	FMHSS	0.157	0.19	2.24	0.002	0.004	-	0.46	-	0.022	0.006	0.011	0.001
VA1000_TM05	FMHSS												
VA1200_MTM	FMHSS												
VA1400_TM	FMHSS												
VA1400_MTM	FMHSS												

ASS—austenitic stainless steel; FSS—ferritic stainless steel; FMHSS—ferritic–martensitic high-strength steel.

**Table 2:** The elongation to fracture, yield stress (YS), ultimate tensile strength (UTS), hydrogen sensitivity parameter (HSP), and tensile toughness obtained from constant extension rate tensile testing (CERT). Note: three separate batches of VA1400_MTM steel grade were studied [13].

#	Steel	Elongation to Fracture (%)		YS (MPa)	UTS (MPa)	HSP (%)	$K_{Ic}$ $\;(MJ\;m^{-3})$	$K_{IH}$ $\left(MJ\;m^{-3}\right)$
		ε	$\varepsilon_{H}$					
1	AISI 304	83.7	32.9	309	745	60.7	558	159
2	AISI 409	33.5	11.3	266	397	66.2	117	30
3	AISI 441	29.1	7.7	309	424	73.3	124	47
4	VA1000 M05	6.2	0.84	1059	1233	86.4	110	24
5	VA1000 TM05	7.01	0.77	955	1024	88.9	86	25
6	VA1200 MTM	8.3	0.78	1119	1347	90.5	125	46
7	VA1400 TM	5.4	0.34	1322	1459	93.7	95	5
8	VA1400 MTM	3.4	0.14	1460	1494	96	69	26
9		4.8	0.17	1387	1626	96.4	103	16
10		6.4	0.13	1113	1391	97.9	120	13
